# Tracheal diverticulum in an asymptomatic male: a case report

**DOI:** 10.1186/1757-1626-1-181

**Published:** 2008-09-24

**Authors:** Ioannis Kokkonouzis, Dimitrios Haramis, Ioannis Kornezos, Hippocrates Moschouris, Stamatis Katsenos, Stauroula Bouchara

**Affiliations:** 1 Pulmonary Department, Army General Hospital, Athens, Greece; 2 Department of Radiology, ≪Tzanio≫ General Hospital, Piraeus, Greece; 3 Department of Radiology, ≪Leukos Stauros≫ General Hospital, Piraeus, Greece

## Abstract

**Introduction:**

An air filled lesion can be a diagnostic dilemma and a careful investigation must be following to clarity any underlining pathology.

**Case presentation:**

A 62-year old male, ex smoker, with a history of chronic cough was examined with helical CT tomography and an air filled lesion was demonstrated at the right paratracheal region at the thoracic inlet. A narrow connection to trachea lumen was also visible, a critical element to establish the diagnosis of ttracheal diverticulosis.

**Conclusion:**

This malformation is a rare anomaly with two types, the congenital and the acquired one. It must be included into the differential diagnosis of any air filled lesion at the thoracic inlet. Computed tomography scans (with thin section and reconstructed images) seem the proper imagine. Bronchoscopy can also visualize the diverticulum although sometimes the connection with trachea can't be detected. In most cases is asymptomatic and needs no special treatment. A possible danger of repeated respiratory infections and insufficient intubation and/or ventilation must be in mind.

## Introduction

An air filled paratracheal lesion is a rare radiological finding and several entities must be included in the differential diagnosis. Although in most cases the physician's suspicion is oriented to the digestive system, some rare anomalies arising from the respiratory system could be the cause. One of these is tracheal diverticulosis. This malformation has two forms-the congenital and the acquired one- and although some patients remain asymptomatic, others may have a history of repeated respiratory infections or unsuccessful intubation.

## Case presentation

**Figure 1:**
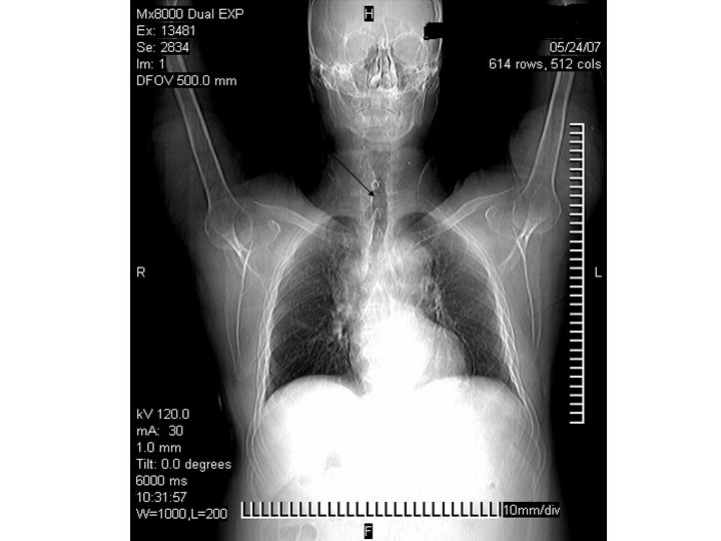
On scanogram the trachea diverticulum is demonstrated on the right side of the trachea at the thoracic inlet.

**Figure 2:**
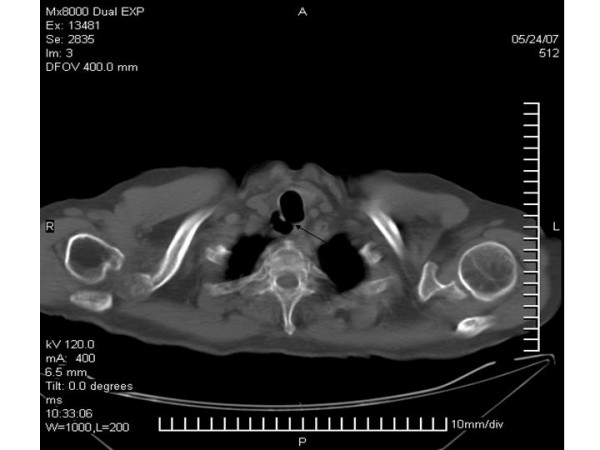
**On CT scan the diverticulosis is demonstrating on the right posterior-lateral region of the trachea. **The connection with the tracheal lumen is also obvious at the posterior wall of the trachea.

A 62-year old male, ex-smoker, with a history of chronic cough was referred for helical chest computed tomography. Until then physical examination, spirometric values and routine laboratory results were unremarkable. On scanogram an ovoid air filled lesion originating from the trachea was detected at the right paratracheal region, at the level of the thoracic inlet (figure 1). The connection with the tracheal lumen was also visible. On computed tomography scans of the chest an ovoid air filled structure was demonstrated on the right posterior-lateral region of the tracheal lumen. No wall thickening or calcifications were found. The presence of a narrow stalk connecting the lesion with the posterior wall of the trachea was critical in establishing the diagnosis of a tracheal diverticulum (figure 2). Another remarkable finding was a 4 cm thoracic aorta's aneurysm. The patient refused further diagnostic work-up with bronchoscopy.

## Discussion

The differential diagnosis of an air filled paratracheal region includes a laryngocele, a pharyngocele, a Zenker's diverticulum, tracheal diverticula, an apical hernia of the lungs and apical paraseptal bullae. Radiological barium swallow study, endoscopic techniques and computed tomography can be used to characterise the lesion [1-3].

Tracheal diverticulum is rare and usually found post-mortem. The frequency of the tracheal diverticulum in some autopsy series has been estimated to 1%. On the other hand, Goo et. al. reported sixty four proven cases in a single study among livings [2,4]. In most cases tracheal diverticulum is located at the right posterior-lateral side of the trachea and it appears to have one or multiple connections with this structure. Two types have been reported; congenital and acquired. Congenital tracheal diverticulum is smaller, located approximately 4–5 cm below vocal cords or just above the carina. It's more common in males than females and can be considered as a supernumerary, malformed branch of the trachea. The histological structure of this diverticulum resembles that of trachea. Other malformations, such as a tracheoesophageal fistula, can coexist. On the other hand, an increase of the tracheal intraluminal pressure caused by chronic cough or obstructive lung disease with emphysema combined with a weakened musculature of the trachea wall, due to repeated respiratory infections, can lead to the acquired form of tracheal diverticulum. This is larger than the congenital one and its wall consists only of respiratory epithelium, without any cartilaginous or smooth muscular elements. The association of marked dilatation of the trachea and main bronchi, combined, sometimes, with tracheal diverticulum, bronchiectasis and recurrent lower respiratory tract infections is called Mounier-Kuhn syndrome [1-7].

Tracheal diverticulum is commonly asymptomatic and incidentally detected. Nevertheless, it can act as a cavity full of secretions and manifest clinically with chronic cough, repeated episodes of respiratory infections, haemoptysis, stridor or dyspnoea. Compression of the vocal cords by the diverticulum can cause dysphonia [2-4,7,8]. Finally tracheal diverticulum can be the underlying cause for unsuccessful intubation or inefficient ventilation [9,10]. Moller et al reported a case of pneumomediastianum as a complication of such a difficult intubation [11].

Computed tomography examination, including thin sections or reconstructed images, is the proper imaging method for the study of tracheal diverticulum. It can demonstrate the connection between the diverticulum and the tracheal lumen and the possible damage of lung's parenchyma as the result of chronic illness. Although bronchoscopy can establish the diagnosis, diverticula with a narrow opening or just a fibrous connection with the trachea can be bronchoscopically missed [1-3,6,7].

Surgical treatment of tracheal diverticulum is rarely advocated. Conservative measures, such as antibiotics, mycolytics agents and physiotherapy are proposed especially in older patients [1,2,5,7].

## Competing interests

The authors declare that they have no competing interests.

## Authors' contributions

All authors were involved in patient's care. IK, IK and HM prepared the manuscript, DH, SB edit and SK coordinated the manuscript. All authors read and approved the final manuscript.

## Consent

Written informed consent was obtained from the patient for publication of this case report and accompanying images. A copy of the written consent is available for review by the Editor-in-Chief of this journal
